# Inebriety—Its Causes, Its Results, Its Remedy

**Published:** 1888-12

**Authors:** 


INEBRIETY: ITS CAUSES, ITS RESULTS, ITS REMEDY. By Franklin D. Clum, M. D., Philadelphia. J. B. Lippincott Co. 1888.

This book is better suited to general rather than professional readers.

Inasmuch as the establishment of an inebriate hospital in the State of Georgia has been for several years, and still is being agitated, we extract the subjoined paragraph:

"Over fifty different hospitals for inebriates have been established in America. More than thirty of this number are in successful operation; the others have changed into insane asylums, water cures, etc. Three large buildings or institutions are practically 'faith cures,' where all physical remedies and means are ignored. Several asylums are called homes for nervous people, to conceal the real cause and thus protect the patients from the supposed stigma of inebriety. Others are literally lodging-houses where the inmates can remain a few days and recover from the effects of spirits. Several places make a specialty of opium cases; in some the treatment is often empirical. In only a few of these hospitals is the disease of inebriety studied and treated on a scientific basis. The others are passing through the ordeal of 'elimination and survival of the fittest,' incident to every new advance of science. In many of the States large public hospitals are projected and awaiting pecuniary aid from the State or from other sources."

